# OGFOD1 is required for breast cancer cell proliferation and is associated with poor prognosis in breast cancer

**DOI:** 10.18632/oncotarget.3683

**Published:** 2015-03-29

**Authors:** Jae-Hwan Kim, Soon-Min Lee, Jong-Hyuk Lee, Sohyun Chun, Byung-Hee Kang, Sojung Kwak, Jae-Seok Roe, Tae Wan Kim, Hyunsoo Kim, Woo Ho Kim, Eun-Jung Cho, Hong-Duk Youn

**Affiliations:** ^1^ National Creative Research Center for Epigenome Reprogramming Network, Department of Biomedical Sciences, Ischemic/Hypoxic Disease Institute, Seoul National University College of Medicine, Seoul, Republic of Korea; ^2^ Department of Pathology, Seoul National University College of Medicine, Seoul, Republic of Korea; ^3^ College of Pharmacy, Sungkyunkwan University, Suwon, Republic of Korea; ^4^ Department of Molecular Medicine and Biopharmaceutical Sciences, Graduate School of Convergence Science, Seoul National University, Seoul, Republic of Korea

**Keywords:** OGFOD1, G2/M phase, cell cycle, breast cancer

## Abstract

2-oxogluatrate and Fe(II)-dependent oxygenase domain-containing protein 1 (OGFOD1) was recently revealed to be a proline hydroxylase of RPS23 for translational termination. However, OGFOD1 is nuclear, whereas translational termination occurs in the cytoplasm, raising the possibility of another function of OGFOD1 in the nucleus. In this study, we demonstrate that OGFOD1 is involved in cell cycle regulation. OGFOD1 knockdown in MDA-MB-231 breast cancer cells significantly impeded cell proliferation and resulted in the accumulation of G1 and G2/M cells by decreasing the mRNA levels of G1/S transition- and G2/M-related transcription factors and their target genes. We also confirmed that OGFOD1 is highly expressed in breast cancer tissues by bioinformatic analysis and immunohistochemistry. Thus, we propose that OGFOD1 is required for breast cancer cell proliferation and is associated with poor prognosis in breast cancer.

## INTRODUCTION

The superfamily of 2-oxoglutarate (2OG)-dependent oxygenases has recently been highlighted as significant regulators of transcription factors and chromatin modifiers, catalyzing the hydroxylation of proteins and demethylation of histones and nucleic acids [1]. As chromatin regulators, Jumonji (JMJ) domain-containing proteins lead the demethylation of lysine residues in histones to regulate chromatin dynamics [2]. Three isoforms of ten-eleven translocation (TET) are responsible for converting 5-methyl-cytosine (5mC) to 5-hydroxymethyl-cytosine (5hmC) in DNA [3]. As transcription regulators, 2 types of proline and asparagine hydroxylases—PHD and FIH—inactivate the transcriptional activity of hypoxia-inducible factor (HIF) [4].

2-oxoglutarate and Fe(II)-dependent oxygenase domain-containing protein 1 (OGFOD1) has a highly conserved 2OG oxygenase domain [1]. Tpa1p, a homolog of OGFOD1 in *Sacchromyces cerevisiae,* was identified as part of an mRNP complex that influences translational termination [5], and human OGFOD1 was also implicated as a stress granule protein that stalls translation under stress conditions [6]. Consequently, 3 groups recently determined OGFOD1/Sudestada1/Tpa1p to be proline hydroxylases for Rps23 in humans, Drosophila, and *S. cerevisiae* [7-9]. This enzymatic activity governs mRNA translation through the hydroxylation of proline residue in Rps23, a small ribosome-binding protein.

Other functions of OGFOD1 homologs have been reported. Ofd1, a *Schizosaccharomyces pombe* homolog of OGFOD1, has not been found to have oxygenase activity, but it accelerates degradation of the transcription factor Sre1 [homolog of sterol regulatory element-binding protein (SREBP)] through an oxygen-sensitive mechanism [10]. In addition, human OGFOD1 is involved in ischemic cell survival [11]. OGFOD1 transcript and protein levels are high in the serum of patients with chronic lymphocytic leukemia (CLL) [12], indicating that OGFOD1 participates in tumorigenesis. These observations implicate an unidentified function of OGFOD1, particularly in tumorigenesis.

In this study, we demonstrate that OGFOD1 knockdown in breast cancer cells inhibits cellular proliferation and triggers severe G2/M arrest. Specifically, we found that G1- and G2/M-related transcription factors are significantly downregulated by microarray. We also confirmed that OGFOD1 is highly expressed in breast cancer tissues. These findings suggest that overexpressed OGFOD1 stimulates the cell cycle in breast cancer formation.

## RESULTS

### OGFOD1 knockdown impedes proliferation

In mammals, there are 2 isoforms of OGFOD: OGFOD1 and OGFOD2. We subcloned OGFOD1 and OGFOD2 into mammalian expression vector and transfected HA-tagged OGFOD1 and OGFOD2 constructs into HeLa cells. OGFOD1 localized primarily to the nucleus, whereas OGFOD2 was expressed in the cytosol and nucleus (Supplemental Fig. S1A and S1B). We confirmed that endogenous OGFOD1 resided primarily in nucleus by confocal microscopy (Supplemental Fig. S1C).

**Figure 1:**
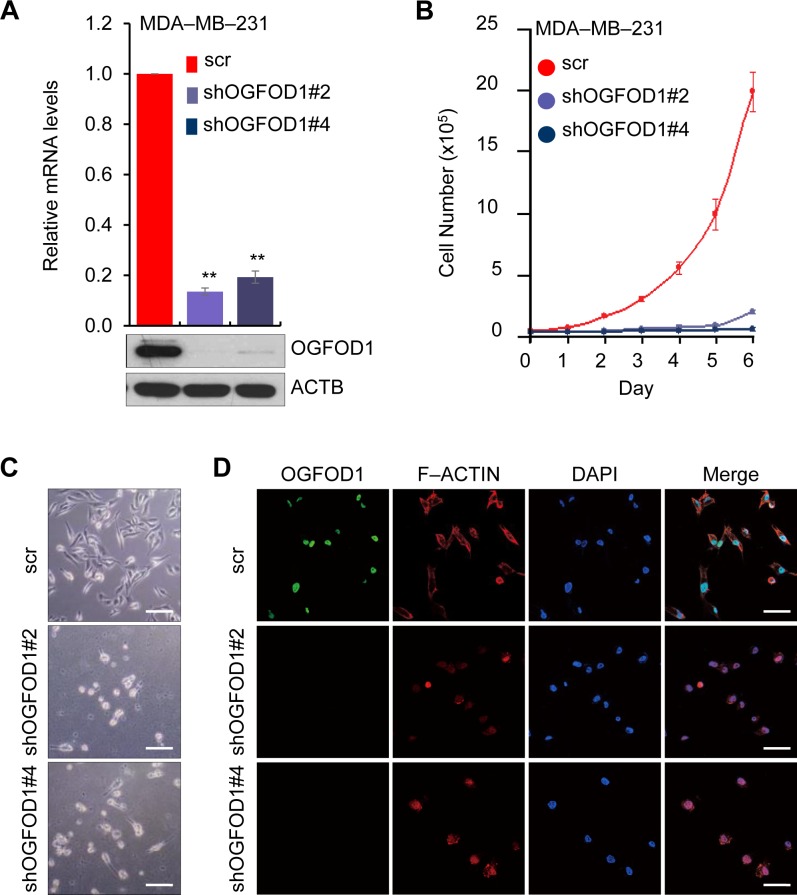
OGFOD1, a nuclear protein, correlated with cell proliferation (**A**) Knockdown efficiency of OGFOD1 shRNAs was examined by RT-qPCR (top panel) and western blot analysis (bottom panel) in the MDA-MB-231 breast cancer cell line. *P*-value was calculated by student *t*-test (*P* < 0.001). (**B**) Effect of OGFOD1 knockdown on cell proliferation in MDA-MB-231 cells. Cells were counted at the indicated time points for 6 days. Data are presented as mean ± SD (error bars) of 3 independent experiments. (**C**) Phase contrast microscopy showing the morphology of OGFOD1 knockdown MDA-MB-231 cells. (**D**) Morphology of OGFOD1 knockdown MDA-MB-231 cells by confocal microscopy. Cells were stained with anti-OGFOD1 (green) and F-ACTIN (red). Nuclei were stained with DAPI (bars = 50 μm).

To determine the function of OGFOD1, we first knocked down OGFOD1 in MDA-MB-231 breast cancer cells using a lentivirally expressed shRNA system (Fig. 1A). OGFOD1 knockdown significantly impeded cellular proliferation (Fig. 1B). Then, we examined the effects of OGFOD1 knockdown on the morphology of MDA-MB-231 cells (Fig. 1C and 1D, Supplemental Fig. 1D). OGFOD1 knockdown led to a condensed structure of intracellular filamentous actin (F-ACTIN). OGFOD1 knockdown cells were round and reflective by phase contrast microscopy and confocal microscopy, which is indicative of living cells in metaphase [13]. These morphological changes in OGFOD1 knockdown cells prompted us to examine the involvement of OGFOD1 in the cell cycle.

### OGFOD1 knockdown results in the accumulation of G1 and G2/M cells

**Figure 2:**
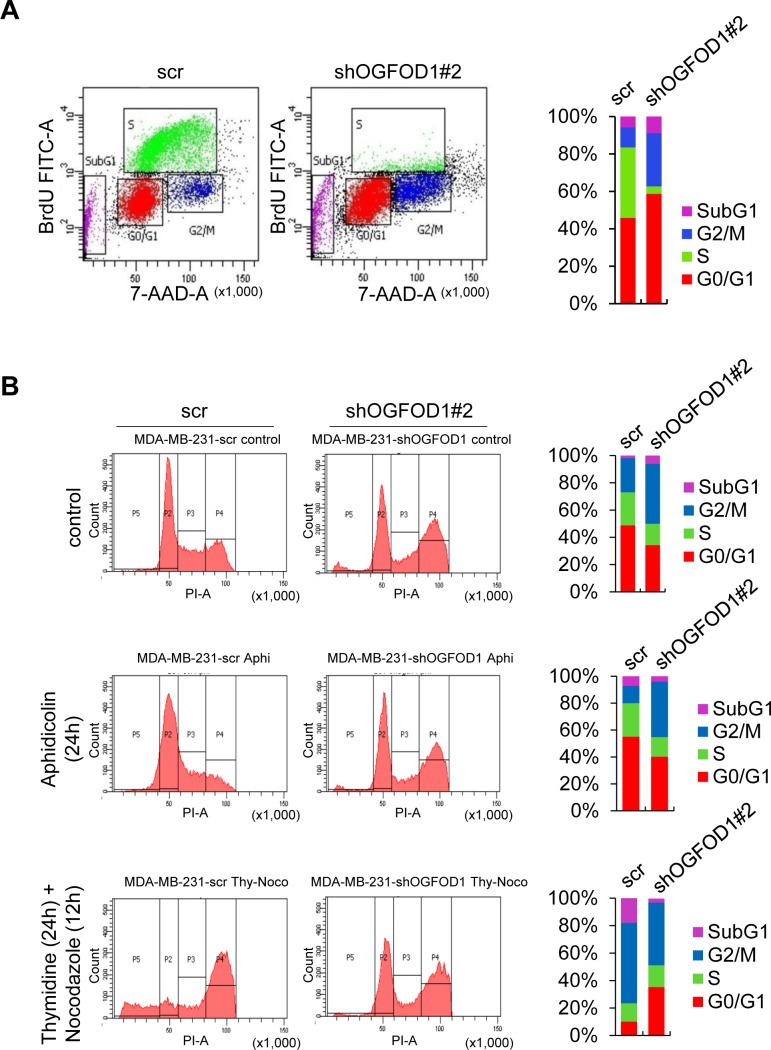
OGFOD1 knockdown leads to accumulation of cells in G1 and G2/M phase (**A**) Double staining of BrdU-FITC and 7-AAD with asynchronous WT and OGFOD1 knockdown cells. (**B**) Cell cycle analysis of OGFOD1 knockdown cells by treatment with aphidicolin (2 μg/ml) for 24 hours or thymidine (2 mM, 24 hours), followed by nocodazole (0.1 μg/ml, 12 hours). Cells were harvested and stained with PI. In both (A) and (B), at least 10,000 cells were collected and analyzed on a Coulter Epics XL™ flow cytometer.

Based on the morphological characteristics of OGFOD1 knockdown cells, we suspected that OGFOD1 might be involved in the cell cycle. Thus, we studied the cell cycle patterns of asynchronous WT and OGFOD1 knockdown MDA-MB-231 cells by BrdU staining (Fig. 2A). Asynchronous OGFOD1 knockdown cells accumulated in G1 and G2/M and absent from S-phase.

To determine the effects of OGFOD1 knockdown on the cell cycle, MDA-MB-231 cells were arrested in G1 phase by treatment with aphidicolin for 24 hours and stained with propidium iodide (PI). OGFOD1 knockdown cells were continuously arrested at G2/M phase, even with aphidicolin, whereas most WT cells were shifted to G1 arrest, indicating that OGFOD1 knockdown blocks the exit from G2/M phase (Fig. 2B).

Next, we treated cells with thymidine/nocodazole to arrest cells at G2/M phase. OGFOD1 knockdown cells were continuously arrested at G1 phase, even with thymidine/nocodazole, whereas most WT cells were shifted to G2/M arrest, indicating that OGFOD1 knockdown inhibits the G1-S transition, consistent with the S-phase deficiency in OGFOD1 knockdown cells. These results indicate that OGFOD1 stimulates the cell cycle genes that are required for G1/S transition and G2/M phase progression.

### OGFOD1 activates cell cycle-related genes

**Figure 3:**
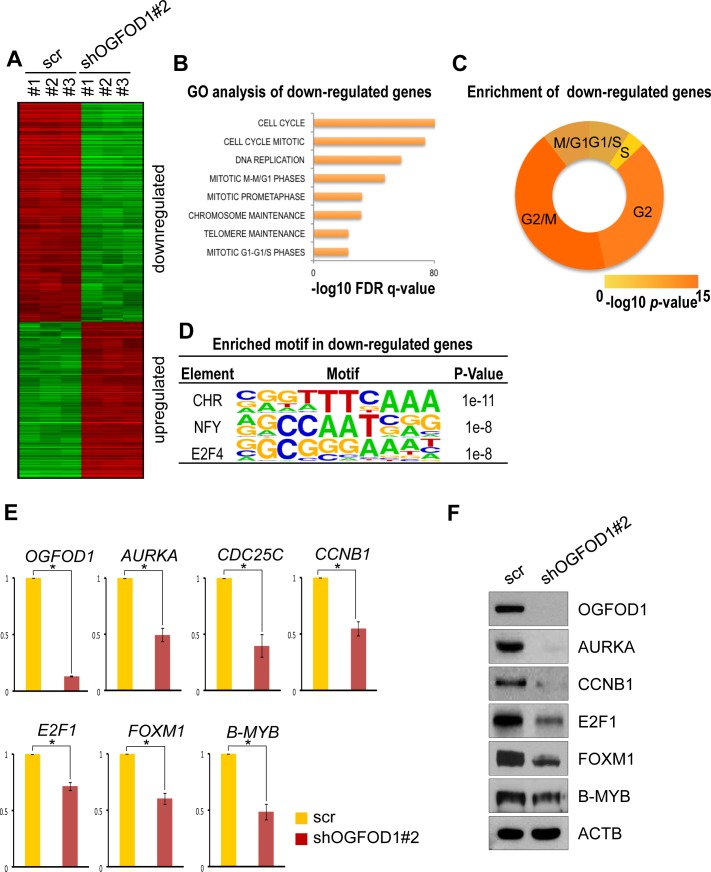
OGFOD1 knockdown reduces the expression of cell cycle genes (**A**) Heat map of the expression of up- and downregulated genes in OGFOD1 knockdown MDA-MB-231 cells compared with WT cells; these genes are differentially expressed genes (DEGs) with 2-fold higher or lower expression. (**B**) Top ranked gene ontologies (biological process) significantly enriched in downregulated DEGs of OGFOD1 knockdown cells. Most gene sets associated with cell cycle regulation. (**C**) Enrichment of phase-specific genes in downregulated DEGs. Genes specific for G2/M phase were most significantly enriched in downregulated DEGs of OGFOD1 knockdown cells. (**D**) Enriched motif in shOGFOD1 downregulated genes. (**E**) RT-qPCR analysis of cell cycle genes in OGFOD1 knockdown cells. (**F**) RT-qPCR analysis of cell cycle-related transcription factors in OGFOD1 knockdown cells. (**G**) Western blot analysis of cell cycle-related genes in OGFOD1 knockdown cells.

To determine the function of OGFOD1 in the cell cycle, we compared gene expression patterns between WT and OGFOD1 knockdown MDA-MB-231 cells by mRNA microarray. We defined up- and downregulated genes as those with at least 2-fold higher or lower expression, respectively (Fig. 3A, Supplemental Table S2). By gene ontology (GO)-based functional analysis, OGFOD1 knockdown led to downregulation of cell cycle-related genes (Fig. 3B). In addition, G1/S- and G2/M (G2)-related genes decreased significantly (Fig. 3C, Supplemental Table S3). By transcription factor binding motif analysis of the downregulated cell cycle genes, we found that downregulated genes in OGFOD1 knockdown cells were predicted to be target genes of cell cycle-related transcription factors, such as CHR, E2F4 [14, 15], and NF-Y [16] (Fig. 3D).

To confirm the expression levels of the cell cycle genes, we measured their mRNA expression by RT-qPCR (Fig. 3E). Consistent with the mRNA microarray results, cell cycle gene expression levels declined significantly in OGFOD1 knockdown cells. Because cell cycle genes are tightly regulated by transcription factors, we examined the mRNA levels of cell cycle-regulating transcription factors. Notably, *E2F1*, *FOXM1*, and *B-MYB* mRNA levels fell in OGFOD1 knockdown cells, consistent with the patterns of cell cycle arrest at G1 and G2/M phase. In addition, we confirmed the reduction in their protein levels in OGFOD1 knockdown cells by western blot (Fig. 3F and Supplemental Fig. S2).

mRNA and protein levels of transcription factors that are essential for G2/M phase, such as *NF-Y* isoforms and components of DREAM complex (*LIN9*/*LIN37*/*LIN52*/*LIN54*), were unchanged in OGFOD1 knockdown cells. Because E2F4 and P130 repress cell cycle genes in G0 phase with DREAM complex [15], we measured the mRNA and protein levels of *E2F4*, other *E2F* isoforms, and *P130* in OGFOD1 knockdown cells; these levels were unaltered in OGFOD1 knockdown cells (Supplemental Fig. S2).

### OGFOD1 knockdown leads to chromatin compaction

The methylation states of H4K20 are tightly regulated during the cell cycle [17, 18]. H4K20me1 declines in G1 phase and accumulates during the S and G2 phases, peaking during M phase. H4K20me3 is highly enriched in transcriptional silencing regions. H4K20me2, in contrast, is broadly distributed throughout the genome.

**Figure 4:**
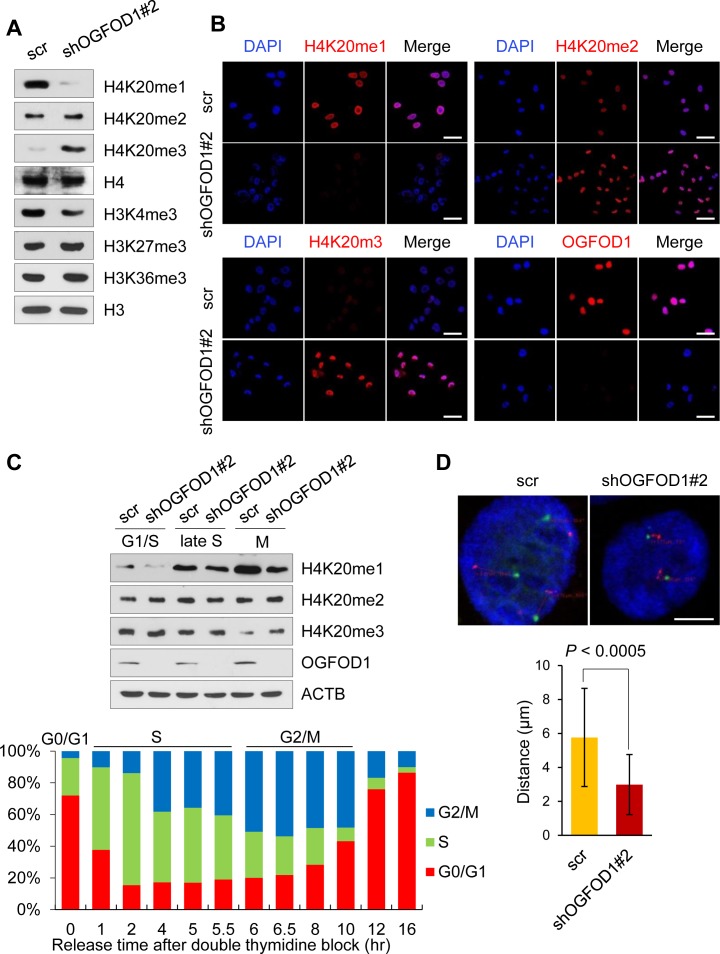
OGFOD1 knockdown results in DNA compaction (**A**) Western blot analysis of methylation states of H4K20 in OGFOD1 knockdown MDA-MB-231 cells. Nuclear fraction was stained with antibodies against methylated H4K20. (**B**) Immunofluorescence staining of methylated H4K20 in OGFOD1 knockdown cells. Mono-, di, and trimethylated H4K20 was stained with the corresponding antibodies (red). Nuclei were stained with DAPI. (**C**) OGFOD1 knockdown cells were synchronized with double thymidine and released by adding serum. At each cell cycle phase (late S; 5 hours, M; 6.5 hours), cells were harvested and probed with the indicated antibodies. (**D**) Measurement of DNA compaction by dual-color fluorescence *in situ* hybridization. Fixed cells were labeled with probes for 16p13.11 (green) and 16q22.1 (red). Distances between 2 probes were measured as described in Materials and Methods. *P*-value was calculated by student *t*-test (*P* < 0.0005).

To determine the link between OGFOD1 knockdown and H4K20 methylation, we examined mono-, di-, and tri-methylated H4K20 in OGFOD1 knockdown MDA-MB-231 cells by western blot (Fig. 4A). Notably, H4K20me3 rose significantly in OGFOD1 knockdown cells, whereas H4K20me1 decreased. We confirmed these findings by confocal microscopy (Fig. 4B). Except for H4K20 methylation, other histone-markers-H3K9me3, H3K27me3, H3K36me3, and H3K27ac-were unchanged in OGFOD1 knockdown cells (Supplemental Fig. S3).

To analyze the effects of H4K20 methylation in OGFOD1 knockdown cells during the cell cycle, we arrested cells at the G1-S border by double thymidine block, released them into normal medium, and harvested them at the indicated phases during the cell cycle (Fig. 4C). In M phase, H4K20me3 levels in OGFOD1 knockdown cells were greater compared with WT cells, consistent with our data that OGFOD1 knockdown triggers G2/M arrest.

Because proliferating cells accumulate high levels of H4K20me1 and little H4K20me3 and because the accumulation of H4K20me3 triggers chromatin compaction [19], we studied this phenomenon in OGFOD1 knockdown cells by dual-color fluorescence *in situ* hybridization with probes for 2 loci on chromosome 16 [19-22]. As a result, the distance between the 2 probes in OGFOD1 knockdown cells was shorter than in WT cells (Fig. 4D), consistent with the western blot data on the accumulation of H4K20me3 in OGFOD1 knockdown cells.

### OGFOD1 induces the transcription of cyclin B1

**Figure 5:**
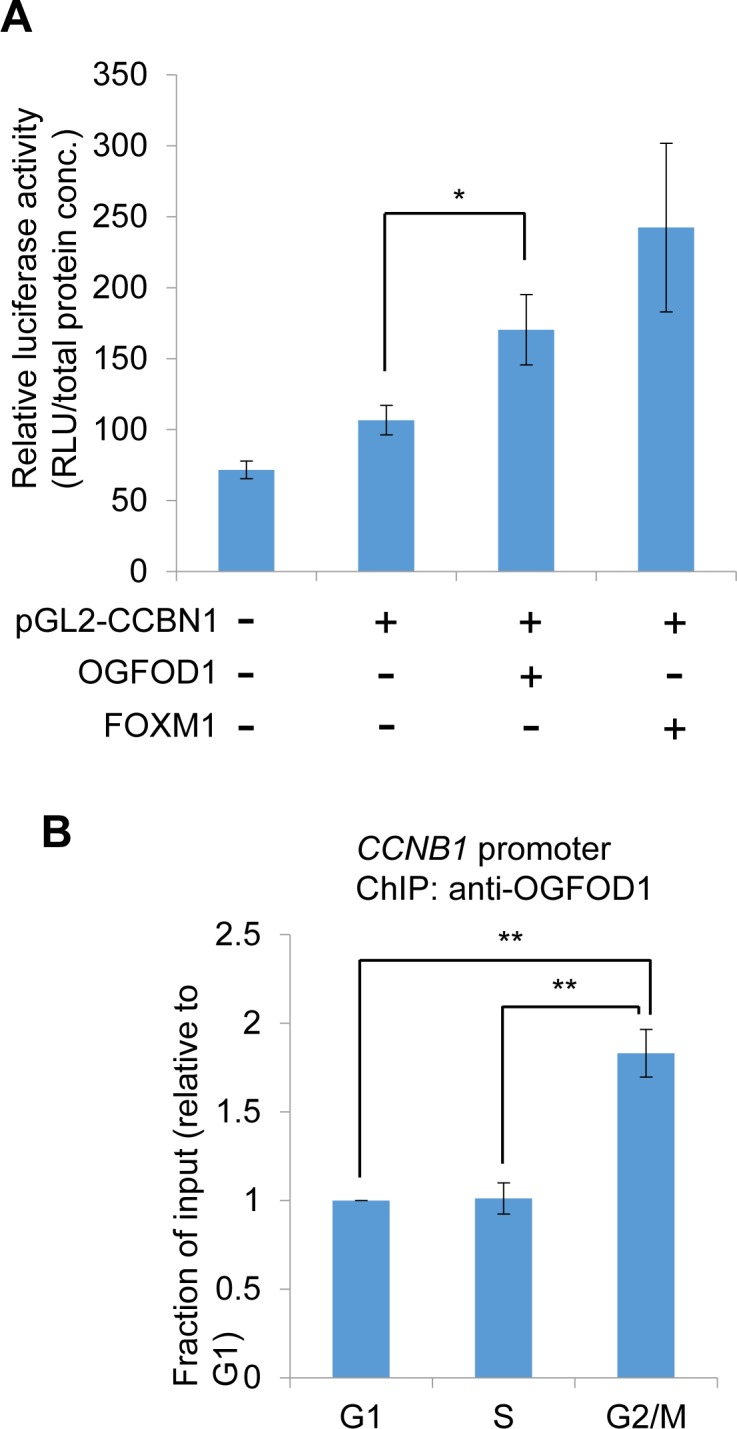
OGFOD1 induces the transcription of cyclin B1 (**A**) Luciferase assay of cyclin B1(*CCNB1*) promoter. Wild-type human *CCNB1* promoter harboring a luciferase construct (pGL2-*CCNB1*) was transfected into 293T cells with/without Flag-OGFOD1 or Flag-FOXM1. Total cell lysates were mixed with luciferase substrate. Relative values are indicated (luciferase unit/total protein concentration). (**B**) Chromatin immunoprecipitation (ChIP) of OGFOD1 on *CCNB1* promoter. MDA-MB-231 cells were synchronized with the indicated cell phase as described in Materials and Methods, and ChIP was performed using OGFOD1 antibody. Real-time qPCR was performed with primers corresponding to the human *CCNB1* promoter. Data are the average values of at least 3 independent experiments. Standard deviations are indicated as error bars. *P < 0.05, **P < 0.001.

Next, we determined whether OGFOD1 correlates with G2/M cell cycle-related genes. Cyclin B1 (*CCNB1*) contains a regulatory subunit of CDC2 kinase and is required for mitotic initiation [23]. We generated a reporter construct that harbors wild-type human *CCNB1* promoter. FOXM1 and OGFOD1 induced luciferase activity, indicating that OGFOD1 induces *CCNB1* promoter activity (Fig. 5A). Also, by chromatin immunoprecipitation (ChIP), we confirmed that OGFOD1 occupancy during G2/M phase rose significantly at the endogenous *CCNB1* promoter (Fig. 5B). Based on these results, we conclude that OGFOD1 controls G2/M progression, in part, by binding the *CCNB1* promoter and inducing its transcriptional activity.

### OGFOD1 is highly expressed in breast cancer cells

**Figure 6:**
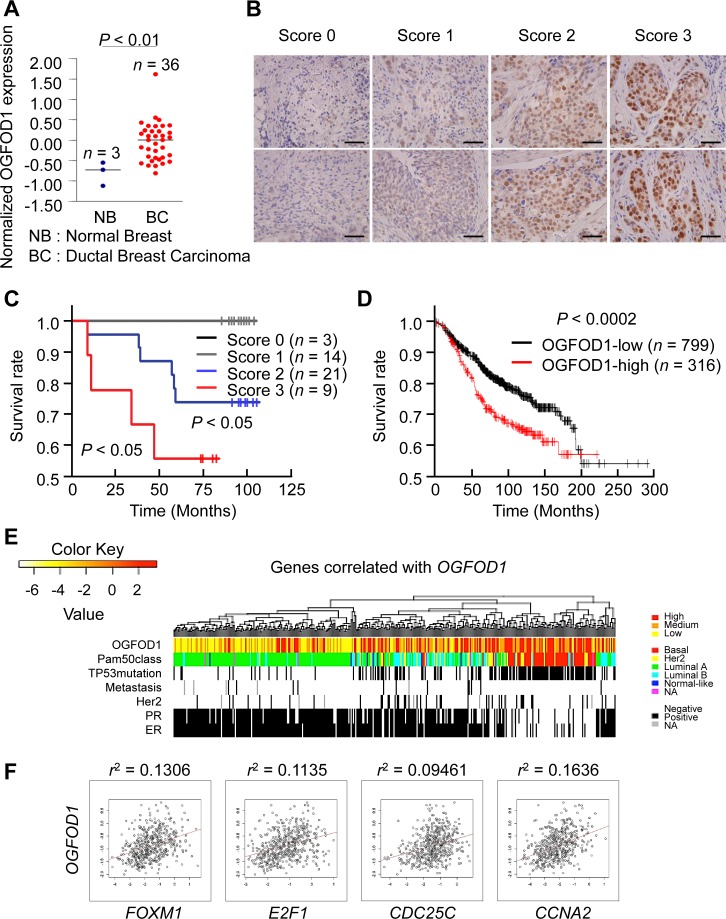
OGFOD1 is highly expressed in breast cancer tissues (**A**) Expression analysis of breast tumor patients from Oncomine dataset [31]. Normal breast tissues (*n*=3) and ductal breast carcinomas (*n*=36) were reanalyzed to show expression of OGFOD1. Bar indicates median. *P*-value was calculated by student *t*-test. (**B**) Representative photographs of immunohistochemical staining of OGFOD1 in breast tumor tissues. Staining intensity was scored 0 (negative nuclear staining), 1 (positive nuclear staining), 2 (moderate nuclear staining), or 3 (strong nuclear staining). Each photograph is shown at x200 magnification. (**C**) Survival analysis of breast tumor patients by OGFOD1 expression level. (**D**) Kaplan-Meier plot of 1115 patients with breast tumors (overall survival) based on OGFOD1 expression. Expression of OGFOD1 was split by upper quartile. The log-rank *P*-value showed a significant difference (*P* < 0.0002). (**E**) OGFOD1 upregulation associated with aggressive breast cancer subtypes. In 522 breast cancer patients (Cancer Genome Atlas), hierarchical clustering of genes that correlated positively with OGFOD1 expression (top 1% in Pearson's correlation) revealed that the upregulation of OGFOD1 occurred primarily in basal type breast cancers, which have a poor prognosis compared with other types. (**F**) OGFOD1 expression correlated with cell cycle genes associated with the G1-S transition and G2/M phase.

To determine the clinical significance of OGFOD1 in breast tumor patients, we analyzed the survival rates of patients by *OGFOD1* expression in several normal versus tumor tissues (Oncomine database) (Supplemental Fig. S4). The expression of *OGFOD1* was significantly higher in cancer tissues versus normal tissues. *OGFOD1* was also highly expressed in breast cancer patients (Fig. 6A). Next, we measured the relative mRNA and protein levels of *OGFOD1* in a nontransformed mammary epithelial cell line, MCF-10A (control cell line) and breast cancer cell lines, MCF-7 / T47D / ZR-75-1 (luminal A, ER^+^/PR^+/–^/HER2^−^), MDA-MB-231 / MDA-MB-468 / BT-20 (triple-negative, ER/PR/HER2^−^), MDA-MB-453 (HER2, ER^−^/PR^−^/HER2^+^). The expression of *OGFOD1* protein (top panel) and mRNA (bottom panel) was significantly greater in breast cancer cell lines compared with MCF-10A cells (Supplemental Fig. S5).

Then, we studied whether OGFOD1 is highly expressed in various types of cancers by immunohistochemistry using tissue array (Supplemental Fig. S6). OGFOD1 was hardly detectable in normal human tissues (top panels), whereas OGFOD1 expression was high in the corresponding cancer tissues, including breast cancer (bottom panels), which is consistent with the overexpression of *OGFOD1* mRNA in the Oncomine database (Fig. 6A, Supplemental Fig. S4).

Based on the staining intensity of representative immunohistochemical photographs of OGFOD1 in breast cancer tissues (Fig. 6B), we performed a survival analysis (Fig. 6C). Low OGFOD1 expression (scored as 0 and 1) did not affect the survival of breast tumor patients, whereas high expression (scored as 2 and 3) correlated significantly with poor survival (*P* < 0.05). Next, we performed Kaplan-Meier survival analysis to determine whether greater OGFOD1 expression was linked to the clinical prognosis of breast cancer patients. Of 1115 human breast cancer tissues, 316 had high OGFOD1 expression (28.3%), whereas 799 expressed low levels of OGFOD1 (71.7%) (*P* < 0.0002) (Fig. 6D). These results demonstrated a close correlation between higher OGFOD1 levels and poor prognosis in breast tumor patients.

To confirm the clinical significance of elevated OGFOD1 in breast cancer, we collected public mRNA microarray data from breast cancer patients and analyzed *OGFOD1* expression with regard to PAM50-defined disease subtypes (Fig. 6E). Notably, patients with high *OGFOD1* expression were highly associated with basal type breast cancer, which is ER-negative and TP53-mutated and linked to a poor prognosis. In addition, major G1 and G2/M cell cycle genes were highly elevated in these groups (Fig. 6F).

In conclusion, we have demonstrated that OGFOD1 stimulates cellular proliferation by influencing cell cycle progression and that highly elevated levels of OGFOD1 promote cell cycle progression and eventually trigger tumor formation in breast cells. We propose that overexpression of OGFOD1 is a marker of poor prognosis in breast cancers.

## DISCUSSION

Although 2OG oxygenases have been implicated as transcriptional regulators and epigenetic regulators in chromatin, few reports on OGFOD1 have been published. Recently, human OGFOD1, Drosophila Sudestada1, and budding yeast Tpa1p were identified as proline hydroxylases for RPS23, which is involved in translational termination [7-9], supporting a previous report that human OGFOD1 increases translational arrest under cellular stress [6]. Nonetheless, it remains unknown how nuclear OGFOD1 is linked to the ribosomal protein RPS23 for translation termination in the cytoplasm.

Moreover, human OGFOD1 is linked to stress granule formation in response to translational stress, but knockdown of Drosophila Sud1 fails to induce stress granule formation [7]. The OGFOD1 complex, purified from human cells, does not contain any significant component of stress granules, indicating that OGFOD1 has distinct functions in the nucleus between various cell types and stages.

In this study, knockdown of OGFOD1 in MDA-MB-231 breast cancer cells significantly reduced cellular proliferation, consistent with a previous report [9]. Based on the morphology of OGFOD1 knockdown cells, similar to metaphase-arrested cells (Fig. 1C and 1D), we suspected that OGFOD1 controls the cell cycle. OGFOD1 knockdown led to the accumulation of cells in G1 and G2/M phase and a decrease in S phase cells; these results were consistent with the microarray data, showing that G2 and G2/M cell cycle genes are primarily downregulated in OGFOD1 knockdown cells.

Further, binding motifs of cell cycle-regulating transcription factors were highly enriched in downregulated genes in OGFOD1 knockdown cells, such as the cell cycle-dependent element and cell cycle homology region (CDE/CHR) and NF-Y (Fig. 3D). CDE/CHR is associated with the maximal transcription of genes in G2 and M phase [14, 15]. DREAM complex (LIN9/LIN37/LIN52/LIN54) bind to the CHR with E2F4/P130 in G0 and G1 phase, but they switch partners (B-MYB and FOXM1) to activate G2 and M phase genes, such as *CCNB1*, *CDC2*, *CDC25C*, and *AURKB.* NF-Y comprises 3 subunits—NF-YA, NF-YB, and NF-YC—and binds to the CCAAT box promoter region in cell cycle genes [16]. CDE/CHR and CCAAT elements are found within the promoters of most cell cycle genes [24]; thus, cooperative binding of these factors in cell cycle gene promoters is necessary for proper cell cycling.

In this study, OGFOD1 knockdown did not reduce the expression of *E2F4* (Supplemental Fig. S2) but downregulated *E2F1*, *FOXM1*, and *B-MYB*, which are transcription factors that mediate G2 and G2/M phase (Fig. 3E). Activator E2Fs (E2F1, E2F2, and E2F3) induce early cell cycle genes during the G1/S transition, whereas E2F (E2F4) in DREAM complex represses the expression of cell cycle-dependent genes in G0 phase [15]. E2F1 also influences G2/M in cooperation with B-MYB [25]. Here we found that OGFOD1 knockdown led to arrest at G2/M phase. Therefore, these findings indicate that OGFOD1 might enhance the activity of these transcription factors by catalyzing their proline hydroxylation. In fact, we observed that OGFOD1 occupancy of the *CCNB1* promoter increased significantly during G2/M phase and induced its transcriptional activity (Fig. 5). Nonetheless, we cannot exclude the possibility that OGFOD1 knockdown might indirectly reduce the transcription of these cell cycle genes. Thus, it is necessary to determine the mechanism by which OGFOD1 governs the expression of other cell cycle-related proteins.

Proper regulation of the cell cycle is critical for cellular proliferation; consequently, dysregulation of the cell cycle has been implicated in the development of cancer. Proliferation-associated genes correlate primarily with cell cycle-regulated genes in tumors, and the proliferation signature in tumors is commonly associated with the expression of G2/M phase genes, such as *MYBL2* (*B-MYB*), *BUB1*, *PLK1* (*polo-like kinase 1*), and *CCNB1* [26]. High expression of B-MYB is linked to breast cancer [27, 28], and increased expression of FOXM1 correlates with a poor prognosis in breast cancers [29]. Further, B-MYB and FOXM1 target genes, such as *CCNB1* and *AURKA*, are associated with breast cancer [27].

In this study, we have demonstrated that *OGFOD1* protein and mRNA levels are high in breast cancer patient tissues (Fig. 6), consistent with data that OGFOD1 knockdown significantly downregulates G2/M-related genes, which are highly expressed in breast cancers. Notably, OGFOD1 correlated with genes that are expressed in ER-positive and TRP53-mutated patients, indicating that it is a marker of poor prognosis in breast cancers.

In conclusion, our results implicate a novel function for OGFOD1 in breast cancers: OGFOD1 stimulates cell cycle progression, and its elevation effects the proliferation of breast cancer cells. OGFOD1 knockdown results in significant arrest in G2/M phase and DNA compaction through the accumulation of H4K20me3 at mitosis. We confirmed that these molecular events are consistent with the high expression of OGFOD1 in human breast tumor tissues by immunohistochemistry and bioinformatic analysis of public microarray data. At this stage, although there is no direct evidence that the enzymatic activity of OGFOD1 is involved in the regulation of the cell cycle, we propose that OGFOD1 is important for cell cycle progression and that its elevation leads to cellular proliferation in breast cancers.

## MATERIALS AND METHODS

### Cell culture

HEK293T, HeLa, MCF10A, MCF7, MDA-MB-453, MDA-MB-468, MDA-MB-231, T47D, BT-20, and ZR-75-1 cells were obtained from ATCC. HEK293T, HeLa, MCF7, MDA-MB-453, MDA-MB-468, MDA-MB-231, and BT-20 cells were cultured in Dulbecco's modified Eagle's medium, supplemented with 10% (v/v) fetal bovine serum and antibiotics. T47D and ZR-75-1 cells were cultured in RPMI-1640 medium, supplemented with 10% (v/v) fetal bovine serum and antibiotics. MCF10A cells were cultured in DMEM-F12 media, supplemented with 10% (v/v) fetal bovine serum, L-glutamine, antibiotics, insulin (20 μg/ml, Sigma), cholera toxin (0.1 μg/ml, Listbiological Labs), hydrocortisone (1 μg/ml, Sigma), and hEGF (0.02 μg/ml, PeproTech). Lipofectamine (Invitrogen) was used to transfect HeLa and MDA-MB-231cells per the manufacturer's instructions.

### Antibodies

Anti-OGFOD1 (HPA003215) and anti-ACTB (A2228) were purchased from Sigma; anti-HA (MMS-101P) was obtained from Covance; anti-LIN54 (A303-799A) was purchased from Bethyl Labs; anti-LIN9 (ab62329), anti-H3K36me3 (ab9050-100), and anti-H4K20me1 (ab9051) /2 (ab9052) /3 (ab9053) were acquired from Abcam; anti-H4 (39269) was purchased from Active Motif; anti-H3 (#9715) was purchased from Cell Signaling; anti H3K27me3 (ABE44) was obtained from Millipore; anti E2F1 (sc-193), anti-E2F4 (sc-866), anti B-MYB (sc-724), anti-NF-YA (sc-10779), anti-P130 (sc-317), anti-FOXM1 (sc-502), anti-CCNA2 (sc-751), and anti-CCNB1 (sc-752) were purchased from Santa Cruz Biotechnology; and anti-AURKA (610938) and anti-AURKB (611092) were purchased from BD Transduction Labs.

### Quantitative real-time PCR (RT-qPCR) analysis of relative mRNA levels

Total RNA was extracted with TriZol^®^ (Invitrogen Life Technologies, Carlsbad, USA) and reverse-transcribed using AMV Reverse Transcriptase XL (Life Science Technologies). mRNA levels were quantified by RT-qPCR with the SYBR^®^ Green qPCR Kit (Finnzymes, F-410L) on the iQ5 and CFX Connect Real-Time PCR Detection System (Bio-Rad) and normalized to 18s rRNA using the 2^−ΔΔCT^ method. The primers are listed in Supplemental Table S1.

### Plasmids

Full-length OGFOD1 and OGFOD2 cDNA was obtained by PCR from the SK-BR-3 breast cancer cell line. Full-length sequences were verified by sequencing analysis. The PCR products were inserted into pcDNA3-HA and pCMV-tag2B for expression in mammalian cell lines.

### RNA interference

Lentiviral vectors that contained the human OGFOD1-targeting sequences pLKO.1-sh-OGFOD1 #1 (TRCN0000038904), #2 (TRCN0000038905), #3 (TRCN0000038907), and #4 (TRCN0000038908) were purchased from Sigma. Empty pLKO.1 vector was used as a control. Lentivirus was produced per the manufacturer's protocol using the BLOCK-iT Lentiviral RNAi expression system (Invitrogen). pLKO.1-sh-OGFOD1 #2 and #4, the most effective constructs, were used in most experiments, unless specifically noted.

### Gene expression analysis

We extracted total RNA from 3 OGFOD1 knockdown and 3 wild-type MDA-MB-231 cell lines using TriZol^®^; these RNA samples were applied to the human HT-12 expression v.4 bead array (Illumina, Inc., San Diego, USA) for gene expression profiling. Gene signal values were log-transformed and normalized by quantile method. Statistical significance was determined by LPE test and fold-change, and the false discovery rate was controlled by adjusting p-values using the Benjamini-Hochberg algorithm. Hierarchical clustering of differentially expressed genes (DEGs) was performed using Cluster 3.0 [30] and visualized through Java TreeView [31]. We performed gene set enrichment analysis using DAVID [32], and the phase-specific genes that were suggested by Grant et al. [33] were compared with downregulated DEGs. We used the Homer program for motif analysis [34].

The expression levels of OGFOD1 were measured in breast cancer patients using data were obtained from Oncomine and the Cancer Genome Atlas [35].

### Cell cycle analysis

For synchronizing scrambled or OGFOD1 knockdown MDA-MB-231 cells into G1 stage, cells were treated with 2 μg/ml aphidicolin (Sigma, A0781) for 24 hours. For G2/M stage, the cells were treated with 2 mM thymidine (Sigma, T1895) for 24 hours. After 3 hours of changes in culture medium, 0.1 μg/ml nocodazole (Calbiochem, 487928) was added for 12 hours. Synchronized cells were stained with propidium iodide (PI, Sigma, P4170). For asynchronous cell cycle analysis, cells were double-stained with BrdU-FITC (BD Pharmingen, 51-2354AK) and 7-aminoactinomycin D (7-AAD, Santa Cruz, sc-221210).

After staining, at least 10,000 cells were collected and analyzed on a Coulter Epics XL™ flow cytometer (Beckman-Coulter). The percentages of cells in each stage were calculated with Multicycle for Windows (Beckman-Coulter). To obtain cells in G1/S, late S, and M phase, cells were treated with 2 mM thymidine for 12 hours, washed twice with PBS, and incubated for 12 hours in fresh culture medium. The cells were then retreated with 2 mM thymidine for 12 hours. Five hours after being released into normal serum, the cells were harvested at 30-min intervals, and cell cycle phases were analyzed on a Coulter Epics XL™ flow cytometer (Beckman-Coulter). Cells that corresponded to late S and M phase were harvested after 5 and 6.5 hours of incubation in fresh culture medium, respectively. Harvested cells were analyzed by western blot.

### Dual-color fluorescence *in situ* hybridization

DNA compaction of scrambled and OGFOD1 knockdown MDA-MB-231 cells was examined using a dual-color fluorescence *in situ* hybridization kit (Cytocell, LPH 022). Cells were spread on a coverslip, fixed, and labeled with probes for 16p13.11 (green) and 16q22.1 (red) per the manufacturer's instructions. Mounted coverslips were visualized and analyzed on an LSM 510 META (Carl Zeiss) confocal microscope. To quantify the distance of the nuclear foci, single-cell images were acquired with no saturated pixels using the same settings. The mean fluorescence intensity in the nuclear chromosomal focus was determined using NIH ImageJ (http://rsb.info.nih.gov/ij/).

Line profiles were obtained from unprocessed images using LSM 510.

### Reporter gene assay

DNA constructs in the luciferase assay were generated by PCR from 293T genomic DNA. The human cyclin B1 (*CCNB1*) promoter, spanning −933 to +133, was amplified and inserted into the pGL2-Basic luciferase vector (Promega). We transfected 293T cells with pGL2-*CCNB1* with Flag-FOXM1 or Flag-OGFOD1. Luciferase activities were measured 48 h after transfection using a TECAN Infinite M200 luminometer (Tecan). Luciferase activities were normalized to total protein concentration.

### Chromatin immunoprecipitation assay

MDA-MB-231 cells (4×10^7^) were harvested and crosslinked with formaldehyde to a final concentration of 1%. The crosslinking reaction was stopped by adding glycine to a final concentration of 0.125 M. The cells were harvested and washed twice with cold PBS, and cytosolic fractions were eliminated with buffer A [5 mM PIPES (pH 8.0), 85 mM KCl, 0.5% NP-40, protease inhibitors]. Nuclear pellets were washed and resuspended in 1X micrococcal nuclease reaction buffer [10 mM Tris-Cl (pH 7.9), 5 mM CaCl_2_, 0.5 mM DTT], and chromatin was digested with micrococcal nuclease (New England Biolabs) and digestion was stopped with EDTA. The remainder of the procedure was performed as described [36].

### Immunofluorescence and confocal microscopy

Cells were grown on coverslips for 16 hours. After transient transfection of OGFOD plasmids, cells were washed 3 times with PBS and fixed with methanol for 30 minutes. After penetration with 0.5% (v/v) Triton X-100 for 30 minutes, the cells were blocked with 2% (w/v) BSA for 1 hour. The cells were then labeled with primary antibody for 1 hour and stained with FITC-conjugated goat anti-mouse and rhodamine X-conjugated goat anti-rabbit (Molecular Probe) for 1 hour. Labeled cells were visualized on an LSM 510 META (Carl Zeiss). The primary antibodies were anti-HA (1:1000, Covance), anti-OGFOD1 (1:1000, Sigma HPA003215), anti-F-ACTIN (1:2000, Abcam ab205).

### Immunohistochemistry

Immunohistochemistry of cancer tissues were performed by SuperbioChips Laboratories (Seoul, South Korea). Briefly, paraffin tissue sections were deparaffinized with xylene and graded ethanol, and antigen retrieval was performed by heating the sections in 10 mM sodium citrate buffer (pH 6.0) at 95°C for 30 minutes. After being washed, the sections were blocked with 2% hydrogen peroxide in methanol at 4°C for 30 minutes. The slides were washed again with distilled water and incubated with horse serum for 1 hour to suppress nonspecific binding. Samples were incubated with anti-OGFOD1 in PBS at 4°C overnight. Signals were visualized with 3,3′-DAB (Sigma). All immunostained sections were counterstained with Mayer's hematoxylin.

### Statistical analysis

Data are presented as means ± standard deviations, and *P*-value was calculated using the student *t*-test calculator (http://www.physics.csbsju.edu/stats/t-test.html). A value of *P* < 0.05 was considered to be statistically significant. All data are a representative of at least 3 independent experiments.

## SUPPLEMENTARY MATERIAL FIGURES AND TABLES








