# Supplementary material for: OGFOD1 is required for breast cancer cell proliferation and is associated with poor prognosis in breast cancer
Source: Oncotarget. 2015 Mar 29;6(23):19528–41. doi: 10.18632/oncotarget.3683 (PMC4637303; doi:10.18632/oncotarget.3683)
Supplement: Supplementary file 1 [file oncotarget-06-19528-s001.pdf]

OGFOD1 is required for breast cancer cell proliferation and is associated with poor prognosis in breast cancer

Supplementary Material

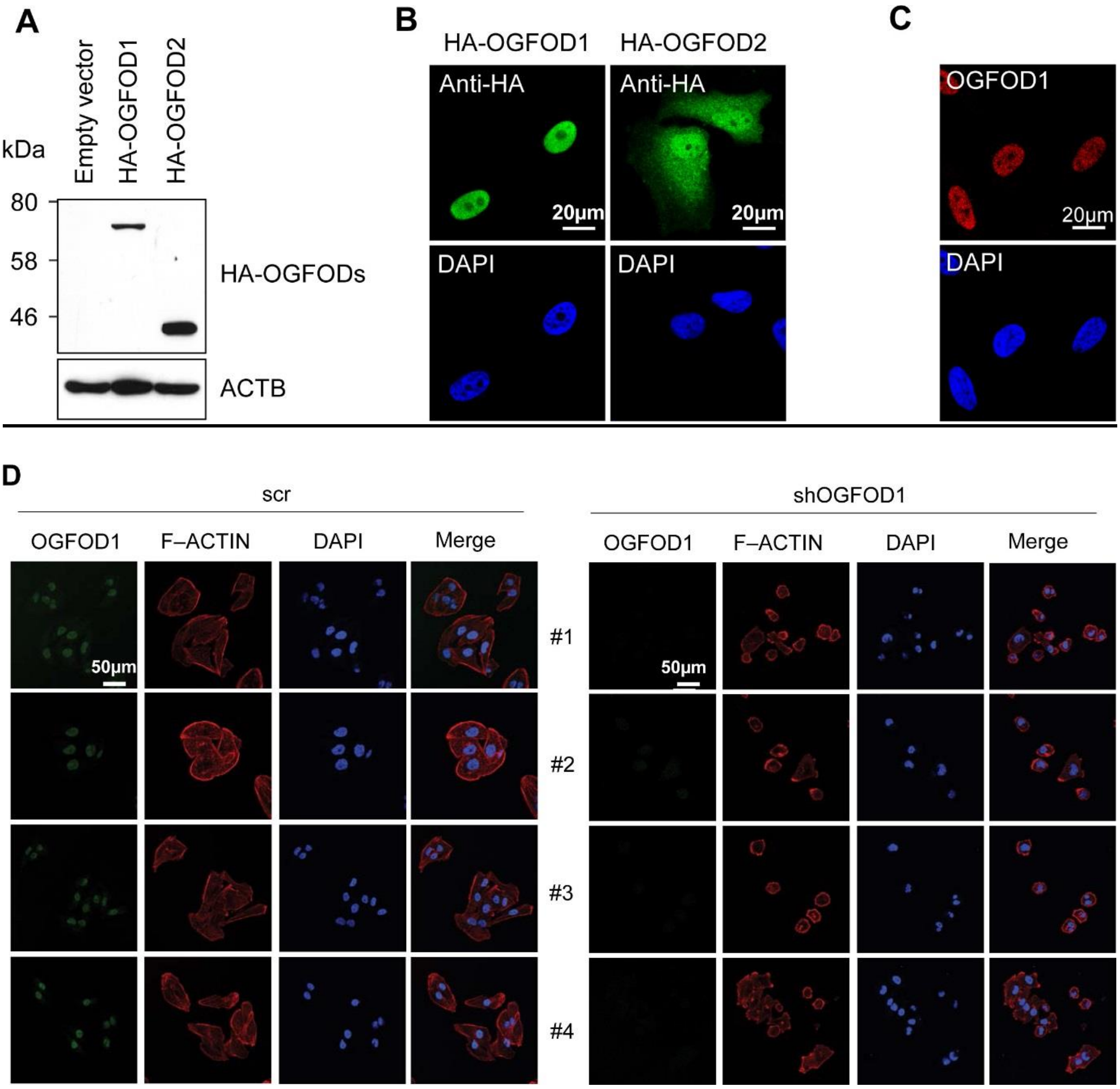

Figure S1: OGFOD1 is a nuclear protein. A, Protein expression levels of HA-tagged OGFOD1 and OGFOD2. 20 μg of whole cell extract was resolved in 12% SDS-PAGE and immunoblotted with anti-HA antibody. Empty vector was transfected as a control. B, Cellular localization of overexpressed OGFOD1 and OGFOD2. Full-length HA-tagged OGFOD1 and

OGFOD2 were transiently expressed in HeLa cells. Cells were then stained with anti-HA antibody (green, labeled with FITC). (Bars = 20 μm) C, Cellular localization of endogenous OGFOD1 stained with anti-OGFOD1 antibody (red, labeled with Rhodamine-red X). Nuclei were stained with DAPI. Staining was analyzed by Zeiss LSM 510 laser scanning microscope. (Bars = 20 μm) D, Morphology of OGFOD1-knockdown MBA-MB-231 cells by 4 different lentivirally-expressed shRNA using confocal microscopy. Cells were stained with anti-OGFOD1 (green) and F-actin (red). Nuclei were stained with DAPI. (Bars = 50 μm).

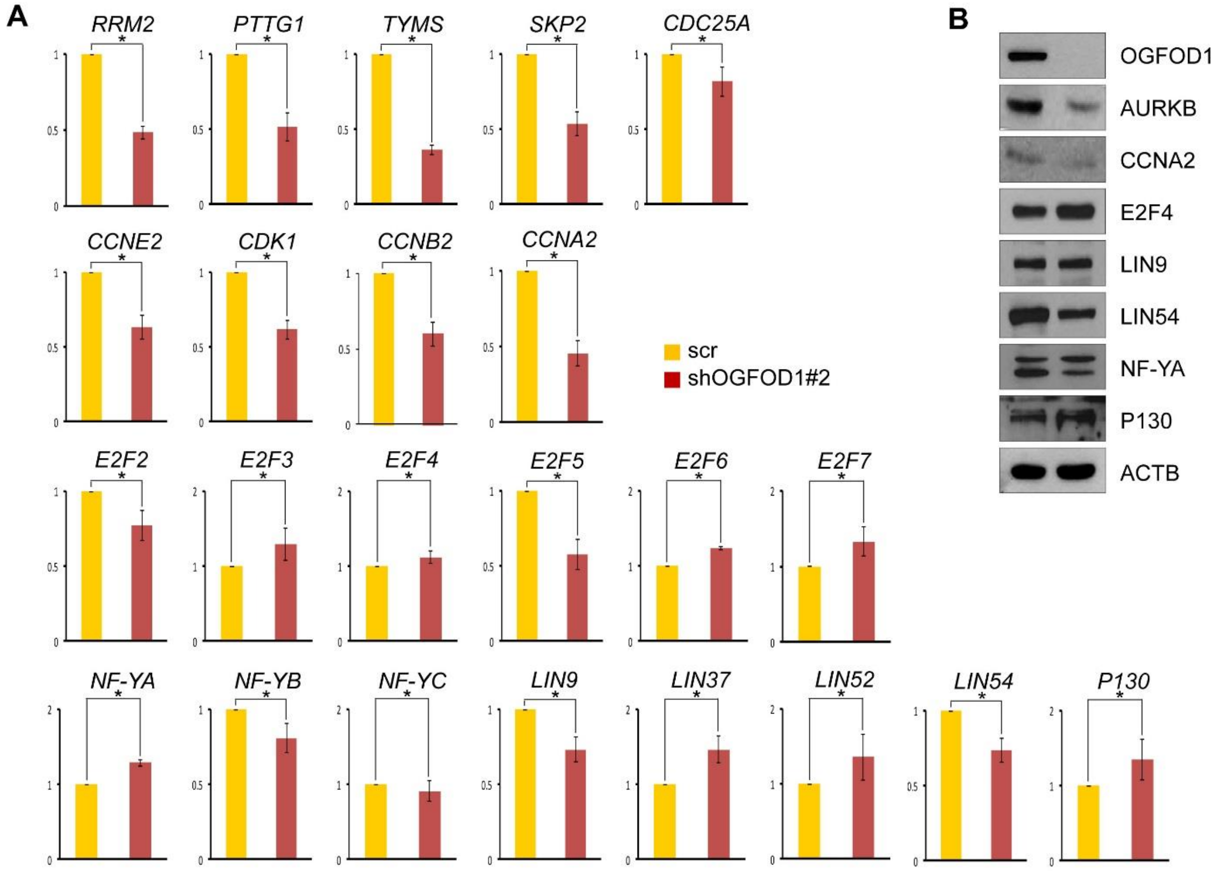

Figure S2: A, RT-qPCR analysis of mRNA levels of cell cycle genes and transcription factors related with cell cycle in OGFOD1-knockdown cells. The *P*-values of each genes are *RRM2-P*<0.005, *PTTG1-P*<0.01, *TYMS-P*<0.001, *SKP2-P*<0.005, *CDC25A-P*<0.01, *CCNE2-P*<0.005, *CDK1-P*<0.005, *CCNB2-P*<0.005, *CCNA2-P*<0.005, *E2F2-P*<0.05, *E2F3-P*<0.05, *E2F4-P*<0.005, *E2F5-P*<0.05, *E2F6-P*<0.0001, *E2F7-P*<0.01, *NF-YA-P*<0.0001, *NF-YB-P*<0.01, *NF-YC-P*<0.005, *LIN9-P*<0.01, *LIN37-P*<0.01, *LIN52-P*<0.05, *LIN54-P*<0.005, and *P130-P*<0.05. B, Western blot analysis of cell cycle-related genes in OGFOD1-knockdown cells.

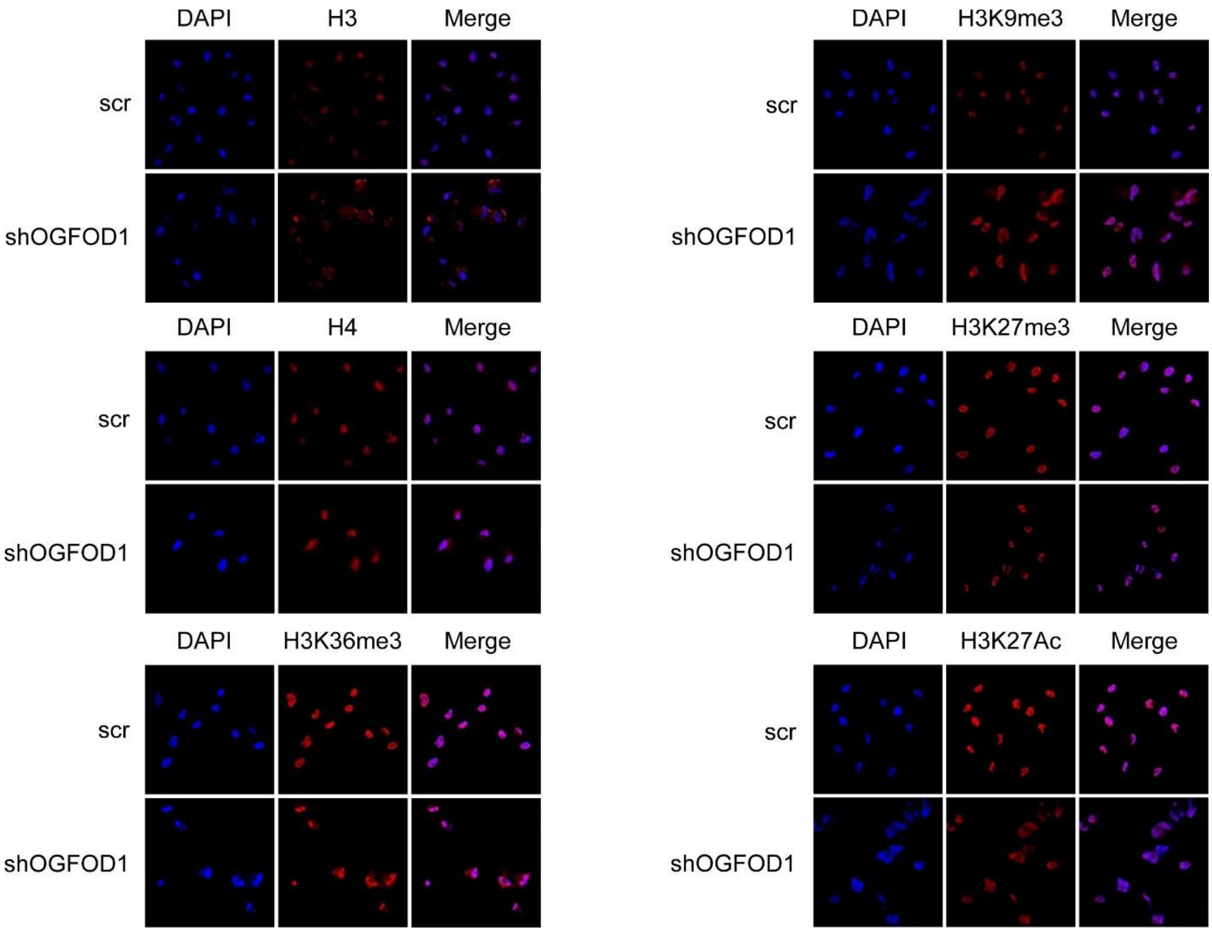

Figure S3: Patterns of various histone post-translational modifications in OGFOD1-knockdown MDA-MB-231 cells. Cells were stained with antibodies recognizing cognate histone PTMs (red, labeled with Rhodamine red-X). Nuclei were stained with DAPI. Staining was analyzed by Zeiss LSM 510 laser scanning microscope.

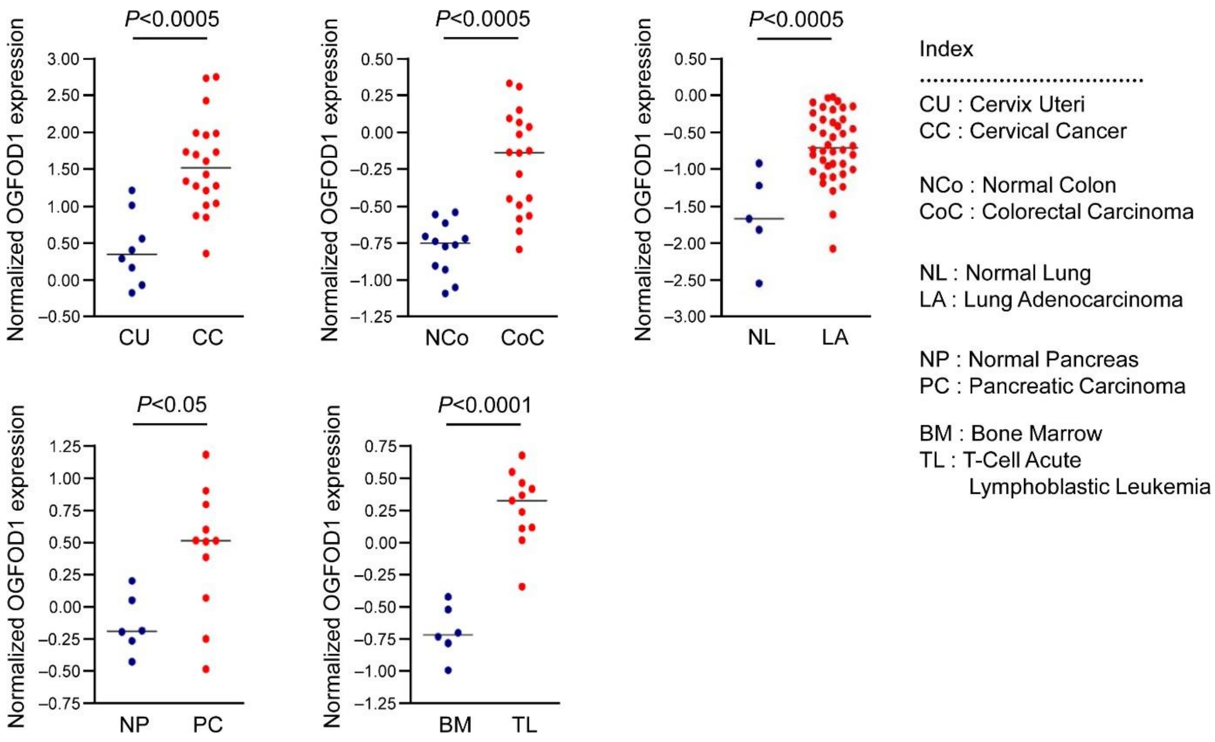

Figure S4: Increase of *OGFOD1* mRNA in various cancer tissues. Comparison of *OGFOD1* mRNA expression in various normal or cancer tissues. *OGFOD1* expression levels from original Oncomine data set were re-analyzed in cervix uteri (CU), cervical cancer (CC), normal colon (NCo), colorectal carcinoma (CoC), normal lung (NL), lung adenocarcinoma (LA), normal pancreas (NP), pancreatic carcinoma (PC), normal breast (NB), ductal breast carcinoma (BC), bone marrow (BM), T-cell acute lymphoblastic leukemia (TL). Bars indicate medians. *P*-values of Student *t*-test were calculated.

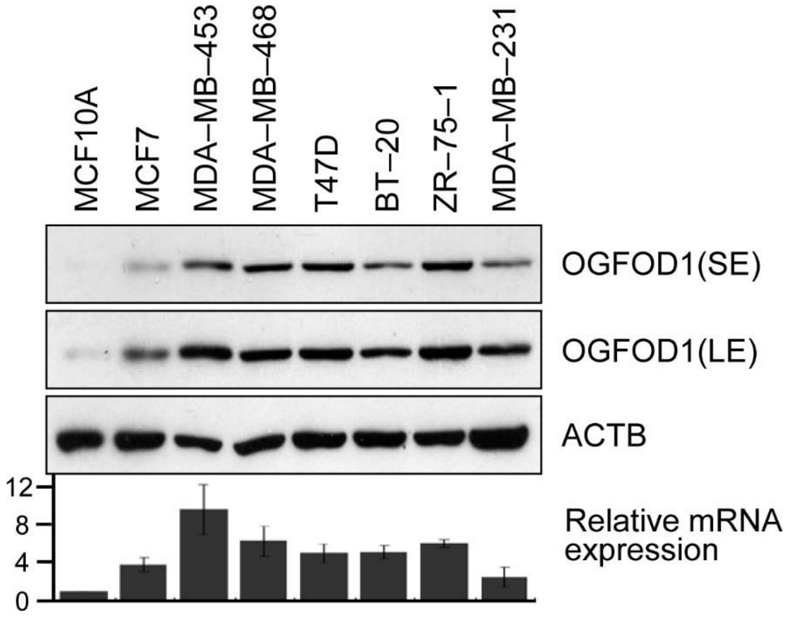

Figure S5: Increase of *OGFOD1* mRNA and protein in breast cancer cell lines. OGFOD1 expression levels (top panel) in immortalized mammary epithelial cells (MCF-10A) and breast cancer cell lines (MCF-7, MDA-MB-453, MDA-MB-468, T47D, BT-20, ZR-75-1, MDA-MB-231). Anti-actin was shown as a loading control. RT-qPCR was performed to compare mRNA expression levels of *OGFOD1* (bottom panel) in each cell lines. Results were normalized with GAPDH. Data are presented as a mean ± SD (error bars) of three independent experiments (*n=3*).

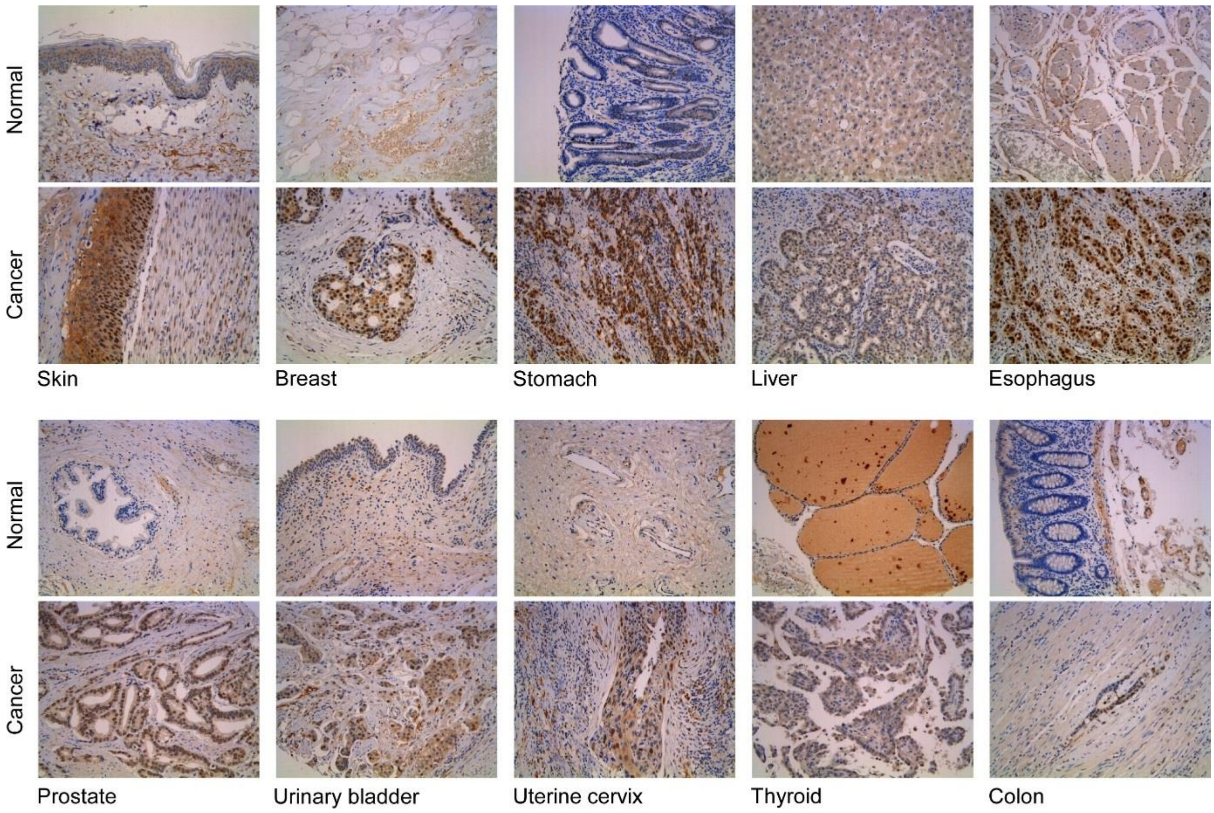

Figure S6: Overexpression of OGFOD1 protein in various cancer tissues. Immunohistochemical staining of OGFOD1 in normal (each top panel) and cancer tissues (each bottom panel) from human skin, breast, stomach, liver, esophagus, prostate, urinary bladder, uterine cervix, thyroid and colon (Tissue microarray). Representative photographs from IHC analysis were shown at x 200 magnification.
